# Phenoloxidase activity and organic carbon dynamics in historic Anthrosols in Scotland, UK

**DOI:** 10.1371/journal.pone.0259205

**Published:** 2021-10-27

**Authors:** Benneth O. I. Esiana, Christopher J. Coates, W. Paul Adderley, Anne E. Berns, Roland Bol

**Affiliations:** 1 Faculty of Natural Sciences, Biological and Environmental Sciences, University of Stirling, Stirling, Scotland, United Kingdom; 2 Graduate School – Research, Aomori Public University, Aomori, Japan; 3 Faculty of Science and Engineering, Department of Biosciences, Swansea University, Swansea, Wales, United Kingdom; 4 Institute of Bio- and Geosciences (IBG-3 Agrosphere), Forschungszentrum Jülich GmbH, Jülich, Germany; 5 School of Natural Sciences, Environment Centre Wales, Bangor University, Bangor, Wales, United Kingdom; Government College University Faisalabad, Pakistan, PAKISTAN

## Abstract

Phenolic compounds are chemical precursor building blocks of soil organic matter. Their occurrence can be inhibitory to certain enzymes present in soil, thereby influencing the rate of decomposition of soil organic matter. Microbe-derived phenoloxidases (laccases) are extracellular enzymes capable of degrading recalcitrant polyphenolic compounds. In this study, our aim was to investigate the relationships between phenoloxidase enzyme activity, organic carbon content and microbial abundance in the context of long-term anthropogenically amended soils. To achieve this, we used a series of complementary biochemical analytical methods including gas chromatography, enzyme assays and solid-state Carbon-13 Cross Polarisation Magic-Angle Spinning Nuclear Magnetic Resonance Spectroscopy (^13^C CPMAS NMR). Using several anthrosols found in St Andrews (Scotland, UK) that had been subjected to intense anthropogenic modification since the medieval period (11^th^ century AD) to present-day, we were able to scope the impact of past waste disposal on soils. The long-term anthropogenic impact led to organic matter-rich soils. Overall, phenoloxidase activity increased by up to 2-fold with soil depth (up to 100 cm) and was inversely correlated with microbial biomass. Solid-state ^13^C NMR characterisation of carbon species revealed that the observed decline in soil organic matter with depth corresponded to decreases in the labile organic carbon fractions as evidenced by changes in the O/N-alkyl C region of the spectra. The increase in phenoloxidase activity with depth would appear to be a compensatory mechanism for the reduced quantities of organic carbon and lower overall nutrient environment in subsoils. By enzymatically targeting phenolic compounds, microbes can better utilise recalcitrant carbon when other labile soil carbon sources become limited, thereby maintaining metabolic processes.

## Introduction

The mechanisms that control carbon (C) dynamics and cycling in soils have received much attention due to their relevance in the global C cycle and being the largest terrestrial reservoir of organic C [1–3]. In natural soils, these mechanisms are generally well-understood, with few uncertainties, such that models of C reserves and fluxes have been developed. In anthropogenic soils, many uncertainties still exist concerning the processes that control C dynamics; this is despite their wide global distribution. Urban areas, particularly historic urban societies, are characterised by having experienced various land-use practices with pronounced ecological heterogeneity. Soils that have developed in urban areas exhibit numerous anthropogenic influences of varying intensity [4] and therefore show distinctness in their properties relative to the natural (or undisturbed) soil. Soils found in these environments reflect the anthropogenic influences they are subject to, including the addition of both inorganic and organic materials. The fate of organic matter applied over time to these soils is controlled by physical (association with minerals), chemical (primary and secondary recalcitrance of organic C compounds) and biological (microbial and enzymatic) modalities. The extent to which soil organic matter (SOM) is stabilised is controlled by combinations of these mechanisms. Biological controls include the nature and capacity of the decomposer community, while the structure of the organic compounds that form the SOM exert physiochemical control. Both are subject to change as decomposer communities adapt, and the structure of SOM will alter over time.

Extracellular enzymes (phenoloxidase (PO), peroxidases) are known to play key roles in the biological mechanism of SOM degradation, including the breakdown of the recalcitrant (poly)phenolic compounds. Phenolic compounds can inhibit the activities of hydrolytic enzymes, through which they may contribute to a slowing down of the degradation of soil organic matter [5]. Unlike hydrolases with well-established interactions and processes in the soil, PO and peroxidase enzyme activity have been measured less frequently, with few studies on anthropogenically altered soils [6]. Given the organic-rich status of historic anthropogenically modified soils, our work investigates the activities of soil microorganisms, PO and peroxidase enzymes, and their role in the mineralisation of SOM. Urban soils are perhaps considered to be a small proportion of soil types in the global inventory—however, their proportion is expected to increase, and therefore, management of SOM in such soils is becoming more relevant in terrestrial systems.

Phenolic compounds represent a chemically diverse, organic grouping of compounds found in soils [6,7]. Natural sources of phenols are derived from plants and by-products of microbial metabolism, whereas anthropogenic sources include agricultural by-products, industrial wastes, and municipal sewage [8–11]. Phenolic compounds are known to contribute to many environmental processes, including the termination of oxidative processes (reactive free radicals to non-reactive stable free radicals) [12,13], SOM degradation, and ultimately suppressing the mineralisation of organic material [11,14–16]. The accumulation of toxic phenolic compounds and their derivatives can modulate the rate of SOM decomposition through non-specific inhibition of hydrolases. Examples of this include lowering soil pH due to the weakly acid hydroxyl/carboxyl groups, chelation of metals (Fe, Al, Cu) by the same chemical groups and forming covalent bonds with amino acids, thereby restricting nitrogen availability and mineralisation [15]. The presence of phenolic compounds in soils, acting in tandem with various other SOM stabilisation mechanisms such as adsorption, aggregation, occlusion etc. [2,17,18] may restrict microbial metabolism and decrease carbon loss from soil, thus increasing the role of soil as a major terrestrial sink for carbon [14].

To counteract the inhibitory and toxic effects of (poly)phenolic assemblages, microbes may synthesize extracellular enzymes, that use either O_2_ or H_2_O_2_ as electron acceptors during catalysis, for example when depolymerizing lignin [6]. Despite the ubiquity of POs in soils, their activities *in situ* are poorly understood [6]. Previously, Freeman et al. [5] investigated the role of phenols and POs in controlling the rate of organic matter decomposition in temperate peatlands. Oxygen availability was found to be a significant restraint on PO activity and, in turn, facilitated the accumulation of intact phenolic compounds that inhibit hydrolytic enzymes (sulphatase, phosphatase, β-glucosidase, chitinase, xylosidase and cellulase). The authors demonstrated that the removal of phenols from soil led to a concomitant increase in the activities of hydrolases. This feedback mechanism was referred to as the ‘enzyme latch’ [5,14]. In contrast, Kang et al. [19] found that in tropical marsh soils, with a different water regime and less organic matter content, a decrease in water level led to a corresponding reduction in enzyme activity and hence an overall decrease in decomposition rate. Positive and negative feedback regulation of PO activities in ecosystems involving varied biotic (e.g., vegetation type) and abiotic (e.g., pH, moisture content, oxygen, temperature, N availability, OM content, litter quality) factors have been reported [15,16].

**Fig 1:**
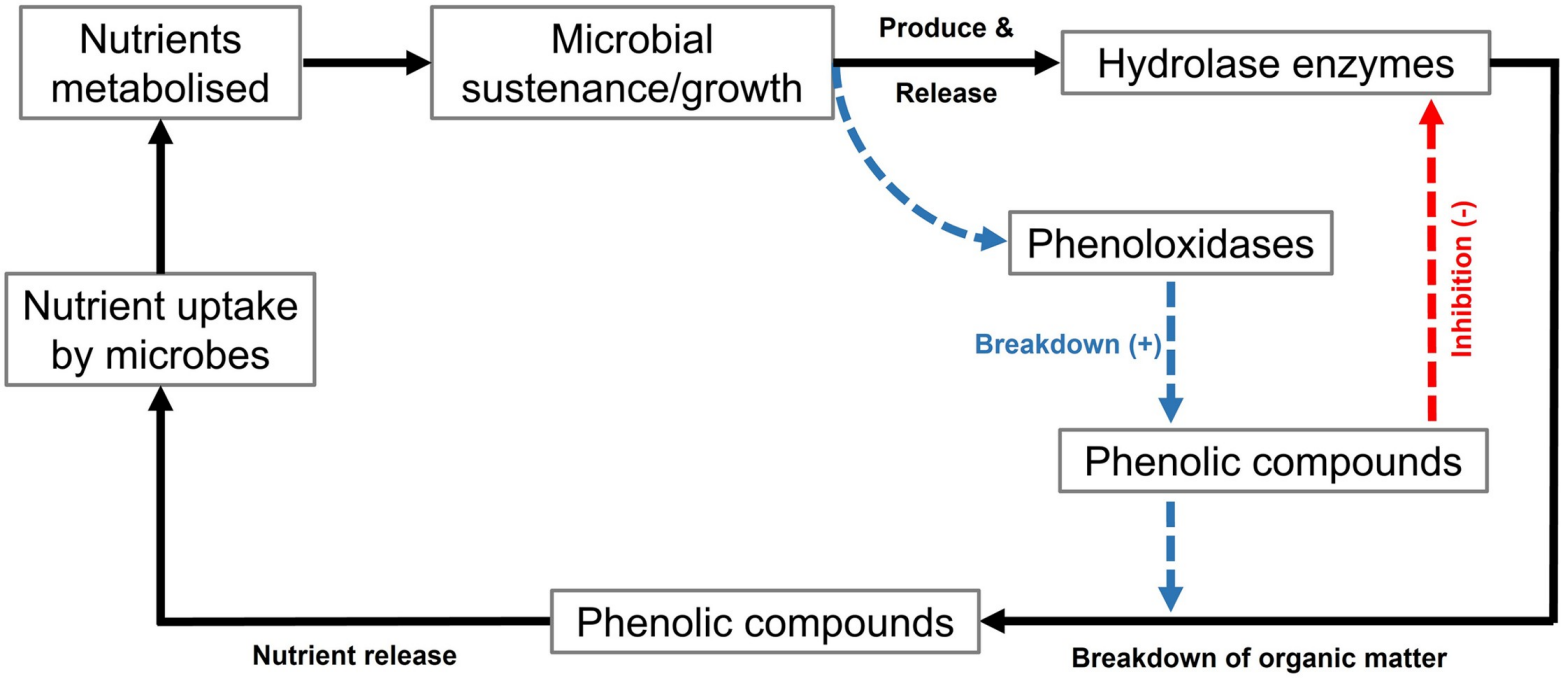
Schematic representation of the role of phenoloxidase enzyme(s) in nutrient cycling. The solid lines represent the normal/primary microbial nutrient cycle; the dotted line represents the secondary nutrient cycle. Plus (+) and minus (-) signs indicate positive (breakdown) and negative (inhibitory) effects on the system.

The present study was designed to investigate the putative relationship between SOM decomposition, PO activity and associated microbial signatures in historic anthropogenically-modified soils. Focussing on Scottish medieval burghs, it was possible to examine sites with extensive multi-century histories of inputs of organic-rich waste material to soils. Historical management practices saw sustained recurrent inputs of diverse organic-rich materials to soils (e.g., bone, food). These additions are natural and anthropogenic sources of phenolic compounds [9,10], which are aromatic structures and likely to have elevated soil phenolic content above the natural background levels, with corresponding associative influences on soil processes (Fig 1). The rate of SOM decomposition varies with depth, generally lower in subsoil profiles and higher in topsoil. SOM content, abiotic conditions, microbial biomass/activity etc. also varies with depth. Our study investigates the implications of such depth-dependent variations on enzyme activity, and the knock-on effect on SOM degradation in anthropogenic soils. PO activity may play a more significant role in SOM degradation at depths relative to the surface profiles. To this end, we addressed the following hypotheses: i) PO enzyme activity will increase with depth in response to lower concentrations of labile organic matter; ii) the presence of phenolic compounds will influence microbial activities and the decomposition of organic matter found in these anthropogenic soils.

## Materials and methods

The St Andrews Preservation Trust Museum and the University of St Andrews (School of History—St John’s House) granted permission to obtain soil samples.

### Study site

**Fig 2:**
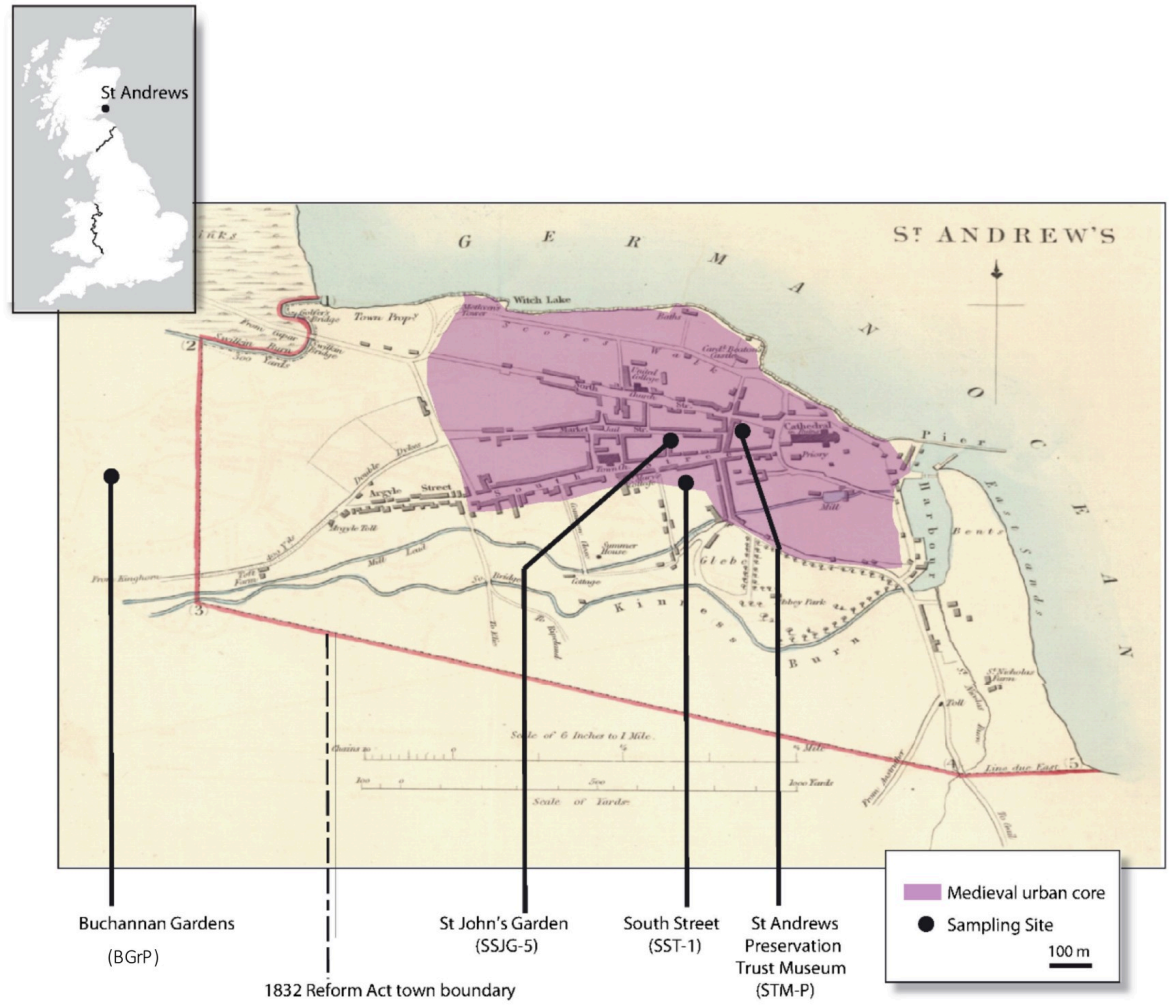
Inset: Location of St Andrews study site in Scotland, UK; Main image: Map of the urban core of St Andrews produced for the Great Reform Act of 1832. The major western and southern boundary is marked as a bold line. Sample site locations in the urban core at St Andrews Preservation Trust Museum (STM-P), St John’s Garden (SSJG-5) and South Street (SST-1) and at an extra-urban location Buchannan Gardens (BGr-P). The original map is reproduced with the permission of the National Library of Scotland (Creative Commons Attribution (CC-BY) licence; https://maps.nls.uk/view/74491935).

Throughout Europe, the medieval period (6^th^ to 15^th^ Centuries A.D.) was characterised by the development of urban settlements and the associated disposal of waste materials (e.g., human and animal faeces, ashes, leather tannery and butchery wastes) by application to the soils found in and around the urban environment [20]. St Andrews, on the east coast of Scotland, is a good representative of this pattern of development. St Andrews became firmly established in the 9^th^ Century AD with a monastic settlement and the late 11^th^ Century saw the establishment of the urban settlement and the town layout that persists to the present-day (Fig 2). The deep anthrosol soils (technic hortic anthrosols) [21] found here today were initially formed during the medieval period with subsequent additions of similar organic material (e.g., bone, food, waste) continuing until at least the 19^th^ Century. The underlying sediments are old red sandstones of Carboniferous age and the immediate surrounding soils are typically podzolic [22]. The present-day land use around the town is arable and improved grassland [23]. The medieval urban areas of St Andrews, where organic-rich wastes were deposited, are today typically gardens and recreational areas dominated by grass cover [24]. St Andrews is representative of other medieval Scottish burghs where sustained additions of waste materials have resulted in deep carbon-rich soils [25–27]. This model of anthropogenic soil formation to form Hortic Anthrosols is seen in many locations worldwide [21]. The depth and extent of these soils increases with proximity to the settlements’ urban centre [28]. The addition of organic waste materials to land in and around this core area continued until at least AD 1832. The practice went into decline in the late 18^th^ century due to urban expansion and new sanitary reforms [29–31]. The Great Reform Act of parliament in 1832 established clear boundaries between the urban core and extra-urban areas (Fig 2).

### Sampling

Sample sites were selected in areas where the medieval town layout remains and where there are minimal contemporary disturbances from modern land-use practices. Thus, undisturbed lawn and garden areas were prioritised. Given the differences in land-use during this period, some variations were expected in the soil organic matter content. Samples were collected from within the medieval settlement. Profiles from St Andrews Preservation Trust Museum (STM-P), South Street (SST-1) and St John’s Garden (SSJG-5), were collected at various points from within the core area (Fig 2) while Buchannan Gardens samples (BGr-P) were collected away from the urban core area. All samples were collected within areas of similar parent material and background soil type.

For all sites, soil bulk samples were obtained from freshly dug and exposed soil profiles. Sampling was conducted on two occasions; initially for physico-chemical analyses (^13^C CPMAS NMR, particle size distribution (PSD)), followed by fresh samples for biochemical analyses (phenoloxidase, and microbial assays). Due to access restrictions arising from a property redevelopment it was not possible to sample soils from South Street site (SST-1) for the biochemical analysis. A total of 33 profile samples were collected and bagged for *ex-situ* analysis. Soil samples for chemical analyses were initially air-dried (60°C) and sieved (< 2 mm); bulk samples for biochemical analyses were stored at 4°C before use.

### Chemical analysis

Total organic carbon (TOC) and nitrogen (TN) were determined by the dry combustion method using an elemental micro-analyser (EA1108 CHNS-O, Carlo Erba Instruments, Milan, Italy). Soil samples (5 g) were oven-dried at 105°C and ground. Aliquots of 20 mg (± 0.20) were weighed into tin capsules and placed in an auto-sampler. Samples were combusted in an oxygen-rich environment (flash combustion) for mineralisation, and elemental composition and content determined by gas chromatography. Blank values were run with empty tin capsules. For calibration, the analysis of a Low Organic Content Soil (B2152) standard with C − 1.26% and N − 0.10% was performed (reference material supplied by the instrument manufacturer). Soil pH was measured in 0.01 M CaCl_2_ (1:2.5 soil:solution ratio) suspension [32] with an HI 209 pH meter (Hanna Instruments, Romania). Approximately, 10 g (± 0.1 g) of air-dry soil was weighed into a beaker containing 25 ml of distilled water. The pH meter electrode was calibrated across pH 4–7 prior to measurement. PSD was determined using a Coulter LS230 series Laser Diffraction Particle Size Analyser utilizing polarization intensity differential scattering based on the Mie theory (Coulter Corporation, Miami, FL, USA) following deflocculation with sodium hexametaphosphate and 30 minutes of mechanical agitation. The measurement range of this apparatus is 0.04 to 2000 μm—performed after a predetermined volume of sample was fed to the instrument (automatically determined by the instrument). The dispersed sample was illuminated by a series of light beams. The particles scatter the light in patterns determined by their respective sizes. A number of photodetectors measured the scattered lights that were scanned and the outputs converted to digital values. Sample particle distribution was categorised using the Wentworth (1922) classification system. Soil moisture content was determined by drying approximately 1g of field-moist soil at 105°C until the mass remains constant.

### HCl/HF treatment

Carbonates due to the presence of ecofact fragments (bone, marine shells), particularly in samples from the urban core area, were removed using a hydrochloric acid (HCl) pre-treatment. Approximately 5 g of 2 mm sieved, air-dried soil was transferred to 50 ml falcon polyethylene centrifuge tubes and 20 ml HCl (12 M) added. The suspension was shaken for 2 hours at 250 rpm at room temperature. Samples were centrifuged and the supernatant removed. The samples were re-suspended in 5 ml of fresh hydrochloric acid and shaken further for 2 hours. On completion, samples were centrifuged and the supernatant removed. Each sample was subsequently washed four times with 25 ml of distilled water to remove traces of HCl until pH 6 was reached. Soils were then dried for 16 hours at 105°C, and ground prior to TOC analysis. A hydrofluoric acid (HF) treatment, modified from Schmidt et al. [33], was carried out on HCl treated samples undergoing ^13^C NMR analysis. This procedure was aimed at improving spectra quality that is otherwise impaired by presence of paramagnetic materials such as iron minerals. Here, 30 ml of 10% HF was added to the 50 ml falcon polyethylene centrifuge tube containing the soil samples. The suspensions were shaken at room temperature for 20 hours, centrifuged for 30 minutes and the supernatant removed. The procedure was repeated four times. The residue was washed five times with deionised water and vacuum filtered using a 0.45 μm cellulose nitrate membrane (Whatman No. 42) to remove salts and any residual HF. Samples were then freeze-dried before analysis. The C:N ratios of samples pre- and post-treatment were determined according to Schmidt et al. [33], [C/N value before treatment]**/**[C/N value after treatment]. HF treatment did not significantly alter the nature of the soil organic matter.

### Soil respiration and microbial biomass

Soil respiration and microbial biomass were determined using the methods detailed by Hopkins and Shiel [34] on the STM-P, SSJG-5 and BGr-P samples. The use of Hopkins and Shiel’s substrate-induced respiration to microbial biomass calibration from their upland field-site in Northern England was considered an appropriate comparator for eastern Scotland. Approximately 5 g of field-moist soil samples was used for analysis. The respiration chambers were incubated at 21°C for a period of 24 hours. The concentration of CO_2_ in a sample of gas from the headspace of the respiration chamber was measured using a Varian aerograph 90-P gas chromatograph (Varian Aerograph, UK) coupled to an NGI Servogor 102 recorder (Cambridge Scientific, Watertown, MA, USA). Respiration rate was calculated using formula 1;

$$\begin{array}{cc} \text{Respiration}\;\text{rate}\;\left(\mu {\text{mol}\;\text{C}\;\text{g}}^{-1}{\;\text{soil}\;\text{h}}^{-1}\right) & =\frac{\left(4.46\text{x}\;10^{-7}\;\times \;\text{Sample}\;\text{peak}\;\text{height}\;\times \;\text{Internal}\;\text{volume}\;\text{of}\;\text{chamber}\right)}{\left(\text{Standard}\;\text{peak}\;\text{height}\;\times \;\text{dry}\;\text{mass}\;\text{of}\;\text{soil}\right)}\\ & =\frac{\left(\text{Formula}\;1\;\text{derivative}\right)}{\left(24\;\text{h}\;\times \;0.27\right)}\;\text{X}\;1000000 \end{array}$$ (1)

Respiration data obtained were assumed to be of microbial origin alone due to the absence of plant remains (roots). Soil microbial biomass was estimated using the regression equation obtained from Hopkins and Shiel [34] to establish a calibration curve.

### Phenoloxidase assay (laccase-type)

Soil samples for phenoloxidase enzyme assays were air-dried, sieved to <2 mm and homogenised using a Retsch MM200 vibrating ball mill (F. Kurt Retsch GmbH & Co. KG, Haan, Germany) [35]. Spectrophotometric detection of ABTS oxidation: Phenoloxidase assays using ABTS (2,20-azino-bis (3-ethylbenzthiazoline-6-sulfonic acid)) as a substrate were modified from Floch et al. [35]. Briefly, 5X modified universal buffer (MUB) was prepared by dissolving 12.1 g of tris (hydroxymethyl) aminomethane (THAM), 11.6 g of maleic acid, 14.0 g of citric acid, and 6.3 g of boric acid (H_3_BO_3_) in 488 ml of a 1 M sodium hydroxide (NaOH) and adjusting the final volume to 1 L using distilled water. For use in enzyme assays, 5X MUB was diluted to 1X using distilled water and adjusting the pH to 3. ABTS solution was made by dissolving 27 mg ABTS in 500 μL of distilled water for a concentration of 0.1 M ABTS. After which, 0.1 g of soil was suspended in 10 ml of MUB solution at a pH 3. A 1 ml representative sample was subsequently removed, and the substrate was added to a final concentration of 2 mM (ABTS). The sample was incubated at 30°C for 5 minutes. After incubation, the sample was centrifuged at 10,500 x *g* for 2 minutes. Supernatant (200 μL) was collected and placed in a single well of a 96-well microtitre plate. Absorbance measurements were recorded at 420 nm using an MDS VERSA max microplate reader (Molecular Devices, Sunnyvale, CA, USA), detecting ABTS^+^ product accumulation. Phenoloxidase activity is expressed as U g^-1^; 1 U is defined as 1 μmol of ABTS^+^ formed per minute, with an absorption coefficient for ABTS^+^ at this wavelength (420 nm) of 18,460 M^-1^ cm^-1^. Assays were performed on three samples per site (three technical replicates per sample) and the results were averaged. Three controls were performed to verify the enzymatic origin of the PO activity detected; A) heat-treated samples (autoclaved at 121°C for 1 h), B) without ABTS added to the reaction mixture and C) without soil added to the reaction mixture.

### Carbon-13 Cross Polarisation Magic-Angle Spinning NMR Spectroscopy (^13^C CPMAS NMR)

Solid-state ^13^C NMR spectroscopy was carried out on HCl/HF treated soil samples to determine the relative proportion of the various organic carbon structures. The ^13^C NMR spectra were obtained on a 7.05 T Varian INOVA^™^ Unity (Varian Inc., Palo Alto, CA, USA) at a ^13^C resonance frequency of 75.4 MHz by applying the cross-polarisation magic-angle spinning (CPMAS) technique. Samples were packed into 6 mm diameter cylindrical zirconia Pencil^®^ rotors (diameter 6 mm) with Vespel^®^ drive tips and spun at 8000 ± 3 Hz in an HX Apex probe. A contact time of 1.5 ms and a 1.5 s recycle delay time were used. Optimal contact times and recycle delays were determined in separate experiments on several samples [36]. During cross-polarization, the ^1^H radio frequency (RF) field strength was set to 48 kHz and the ^13^C RF field strength to 40 kHz. An ascending ramp of 16 kHz on the ^1^H-RF field was used during contact time to account for inhomogeneities of the Hartmann-Hahn condition [37]. The spectra were collected with a sweep width of 25 kHz and an acquisition time of 20 ms. Proton decoupling was done using a SPINAL sequence with a ^1^H field strength of 55 kHz, a phase of 4.5° and a pulse length of 12 μs.

The free induction decays (FID) were recorded with VnmrJ 2.2D (Varian Inc., Palo Alto, CA, USA) and processed by MestRe-C 4.9.9.9 (Mestrelab Research, Santiago de Compostela, Spain). Depending on the organic carbon content of the samples the number of transients collected lay between 40,000 and 250,000 (corresponding to measurement times of 17 to 105 hours per sample). All FIDs were Fourier-transformed with an exponential filter function with a line broadening (LB) of 20 to 50 Hz depending on the sample. Baseline correction was done using the manual baseline correction function of Mestre-C. The chemical shifts are reported relative to tetramethylsilane (= 0 ppm) using adamantane as an external reference. The spectra were divided into five chemical shift regions: carboxyl/carbonyl C (215–160 ppm), aromatic C (160–110 ppm), anomeric C (110–90 ppm), O/N alkyl C (90–45 ppm), and aliphatic C (45-[-10] ppm). The relative intensities of the regions were determined using the integration routine of the MestReC software and subsequently, corrections for the spinning side bands (SSBs) were carried out as described in Conte et al. [36]. The ratio of alkyl C to *O/N*-alkyl C [Eq 2] was calculated and used to assess the degree of decomposition [38–40].


$$\text{Alkyl}\;\text{C}:O/N-\text{alkyl}\;\text{C}=\frac{\text{Alkyl}\;\text{C}\;(0-45\;\text{ppm})}{O/N-\text{alkyl}\;\text{C}\;(45-110\;\text{ppm})}$$ (2)

### Statistical analysis

Preliminary analysis undertaken to identify set(s) of predictors was conducted using best subsets regression in Minitab 17. Pearson’s correlation tests were carried out on selected factors (soil depth and organic C content) with respect to extracellular enzyme (phenoloxidase) activities using GraphPad PRISM v7. *P*-values ≤ 0.05 were used for determining significance. Putative relationships among moisture content, microbial biomass, soil respiration, organic matter content and composition, were evaluated by regression.

## Results

### Relationships between depth and soil variables

**Fig 3:**
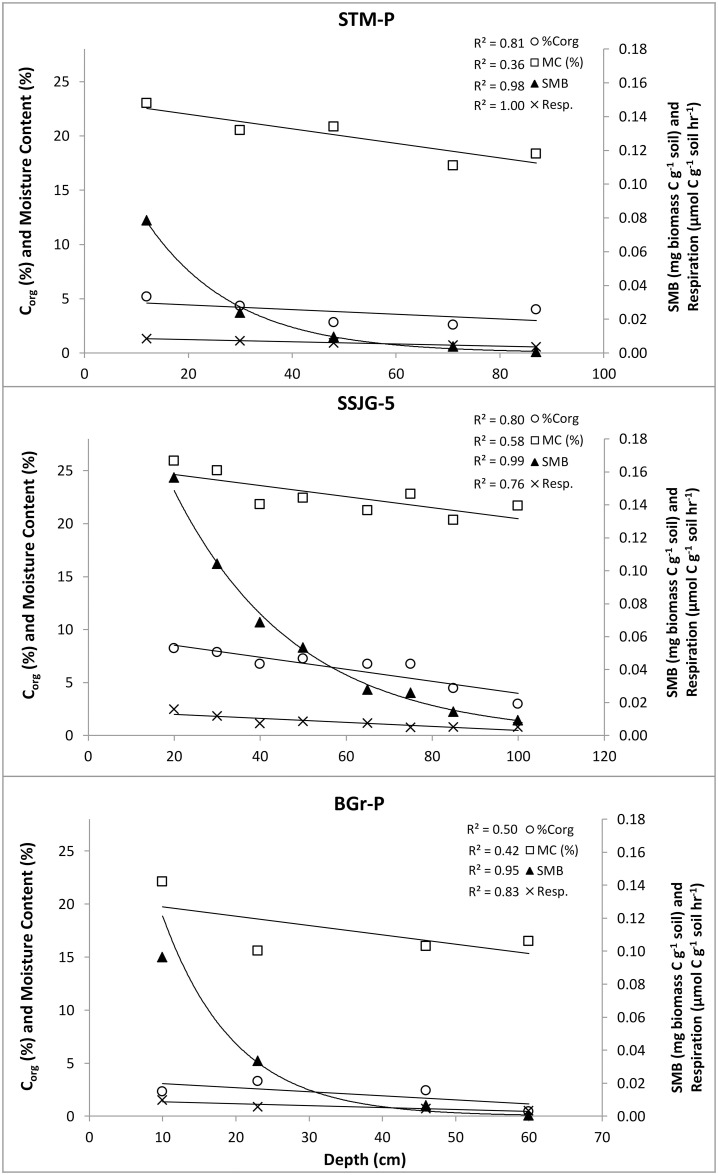
Relationship between soil variables and depth on three sites (STM-P, SSJG-5, and BGr-P). Phenoloxidase enzyme activity was generally higher in subsoils than in topsoil (Fig 4). Enzyme activity was lowest in BGr-P samples for the top 3 depth profiles but similar to values for STM-P and SSJG-5 at subsoil depths, between 40–50 cm. Phenoloxidase activity exhibited a positive correlation with depth on all three sample sites assayed: STM-P, SSJG-5, and BGr-P.

Moisture content, microbial biomass, soil respiration, total nitrogen, and organic carbon content all decreased with increase in soil depth. Total nitrogen values on sites ranged from 0.11 to 0.85%, with SSJG-5 samples dominating the higher range. BGr-P and STM-P share similar low to medium range values. The C/N ratio is lowest on BGr-P (4–9), moderate on SSJG-5 (9–17), and highest on STM-P (12–24); however, no particular pattern or trend was discernible with depth. Soil pH is similarly distributed across all three sites and ranged from 5.48 to 6.06 –which are considered conducive for microbial activity and extracellular enzyme activity (however, no trend was discernible with depth). As expected, the BGr-P site had lower soil organic matter content relative to the other two sites. Moisture content was also lower than observed on the other sites; all other parameters were generally similar. Regression analysis of these variables highlights inverse correlations with depth (Fig 3). Predictably, these factors also show a negative, but variable correlation with phenoloxidase activity (S1 Data).

Soil microbial biomass (SMB) and basal respiration (Resp.) decreased with depth predictably, however, microbial biomass declined exponentially while respiration showed a steady decrease with depth (Fig 3). Low values for microbial biomass observed in STM-P at 71 and 87 cm, and BGr-P at 60 cm are due to the limits of detection and sensitivity of the substrate-induced method because changes in the peak height of CO_2_ evolved were evidently higher in the substrate-induced samples than in the control samples after 24 hours and indicates the presence of microorganisms.

### Phenoloxidase activity

**Fig 4:**
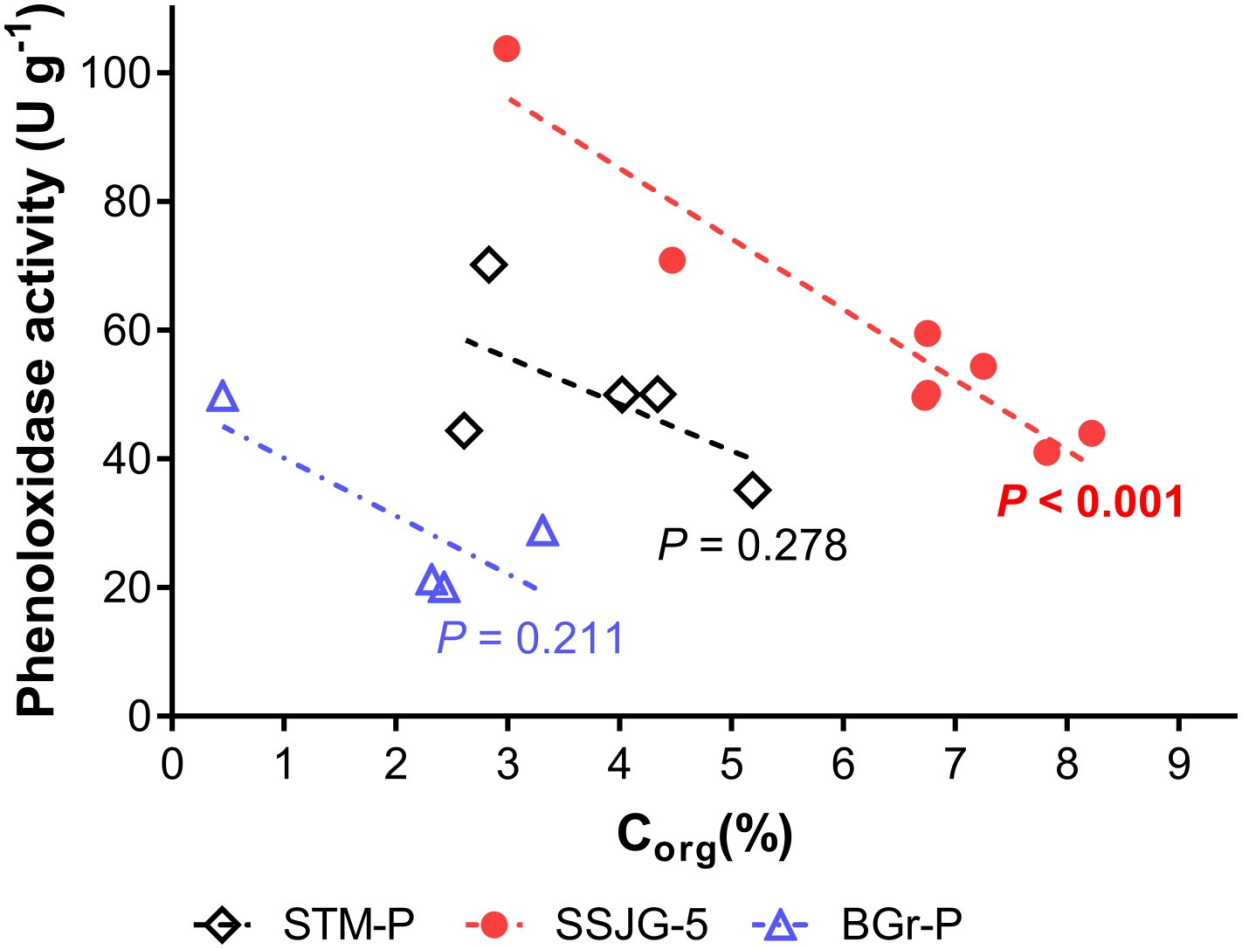
Extracellular phenoloxidase activity versus organic C content across sample sites. STM-P (R^2^ = 0.37), SSJG-5 (R^2^ = 0.91), and BGr-P (R^2^ = 0.62).

**Fig 5:**
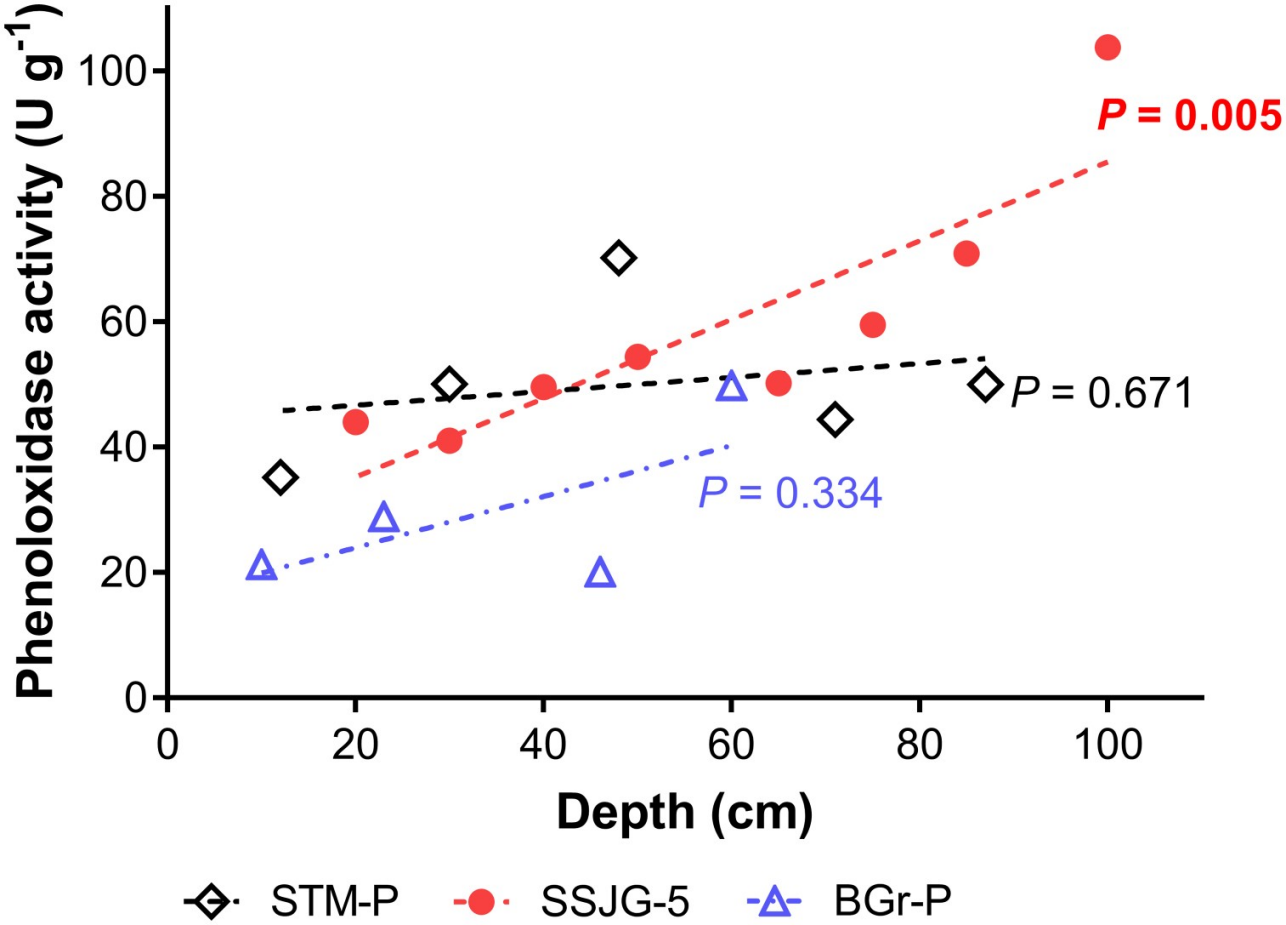
Extracellular phenoloxidase activity versus depth across sample sites. STM-P (R^2^ = 0.068), SSJG-5 (R^2^ = 0.75), and BGr-P (R^2^ = 0.44).

Overall, PO substrate (ABTS) turnover rates were calculated between 20 and 100 U g^-1^ irrespective of the sample origin. Preliminary exploratory analysis by best-subsets regression of PO activity and soil predictor variables revealed that soil organic matter content was the best single predictor variable that accounts for the variation in PO activity observed on all three sites. PO activity correlated inversely with total organic C (TOC) content across all sites (Fig 4; STM-P, *r* = -0.6067, *P* = 0.2780, *R*^*2*^ = 0.39; BGr-P, *r* = -0.7884, *P* = 0.2116, *R*^*2*^ = 0.62), but the relationship was statistically significant for site SSJG-5 (*r* = -0.9518, ***P* = 0.0003**, *R*^*2*^ = 091). Phenoloxidase activity correlated positively with depth on BGr-P (*r* = 0.6657, *P* = 0.3343, *R*^*2*^ = 0.44) and significantly on SSJG-5 (*r* = 0.865, ***P* = 0.0055**, *R*^*2*^ = 0.75), however, this was not as obvious for the STM-P site (*r* = 0.261, *P* = 0.6717, *R*^*2*^ = 0.068; Fig 5).

Three control assays were performed to verify the enzymatic origin of the PO activity detected; A) heat-treated samples (autoclaved at 121°C for 1 h), B) negative control lacking substrate (ABTS) and C) negative control lacking soil in reaction mixture. No oxidative activity was detected in the control assays B and C. In control assay A, substrate oxidation was detected albeit 2 to 4-fold less than in the experimental samples. This result contradicts the report of Floch et al. [35] on the use of autoclave-sterilised samples as a suitable negative control, but agrees with Bach et al. [41] that autoclaving soil does not offer consistent results. The rationale for the use of autoclave treated samples is that enzymes will be denatured at such high temperatures sustained during autoclaving (121°C), however enzymes bound to the mineral matrix of soil are likely to retain functionality across a broad thermal gradient, including extreme cold [42].

### Organic matter content, chemical composition, and particle size distribution

Organic matter content generally declined with depth (Fig 3). Characterisation of carbon species using ^13^C NMR spectroscopy revealed that the observed general decline in total organic matter content corresponded with a general decrease in the *O/N*-alkyl C region in all sites; although, a small rise was detected at a depth of 50 cm on the BGr-P site where there is a twofold increase in clay content relative to surface levels (Table 1). Silt content is also noticeably higher at depth.

**Table 1:** Relative contributions of carbon species to the total signal intensity in ^13^C NMR spectroscopy and particle size fractions of the soil samples.

Sample Location & Sample Depth (cm)		% Alkyl C (0–45 ppm)	% O/N-Alkyl C (45–90 ppm)	% Anomeric C (90–110 ppm)	% Aromatic C (110–160 ppm)	% Carboxyl C (160–220 ppm)	Alkyl C O/N-Alkyl C	Clay (%)	Silt (%)
**Medieval Urban Centre**									
**St Andrews Trust Museum (STM-P)**	0–20	21	15	4	50	11	1.40	3.4	51.7
	20–35	19	12	3	54	11	1.58	3.1	51.4
	35–45	20	11	3	54	12	1.82	4.8	47.1
	45–55	19	10	3	57	11	1.90	5.2	62.9
**St Johns Garden (SSJG-5)**	0–20	23	17	5	43	13	1.35	-	-
	20–35	21	12	4	52	11	1.75	-	-
	35–50	18	10	3	58	11	1.80	-	-
	50–60	20	10	4	56	11	2.00	-	-
**Extra Urban Site**									
**Buchannan Gardens (BGr-P)**	0–20	24	19	4	42	12	1.26	6.6	68.6
	20–30	19	15	4	50	12	1.27	7.3	69.5
	30–40	20	14	5	50	12	1.43	8.4	55.6
	40–50	22	17	4	46	11	1.29	17.7	82.3

**Fig 6:**
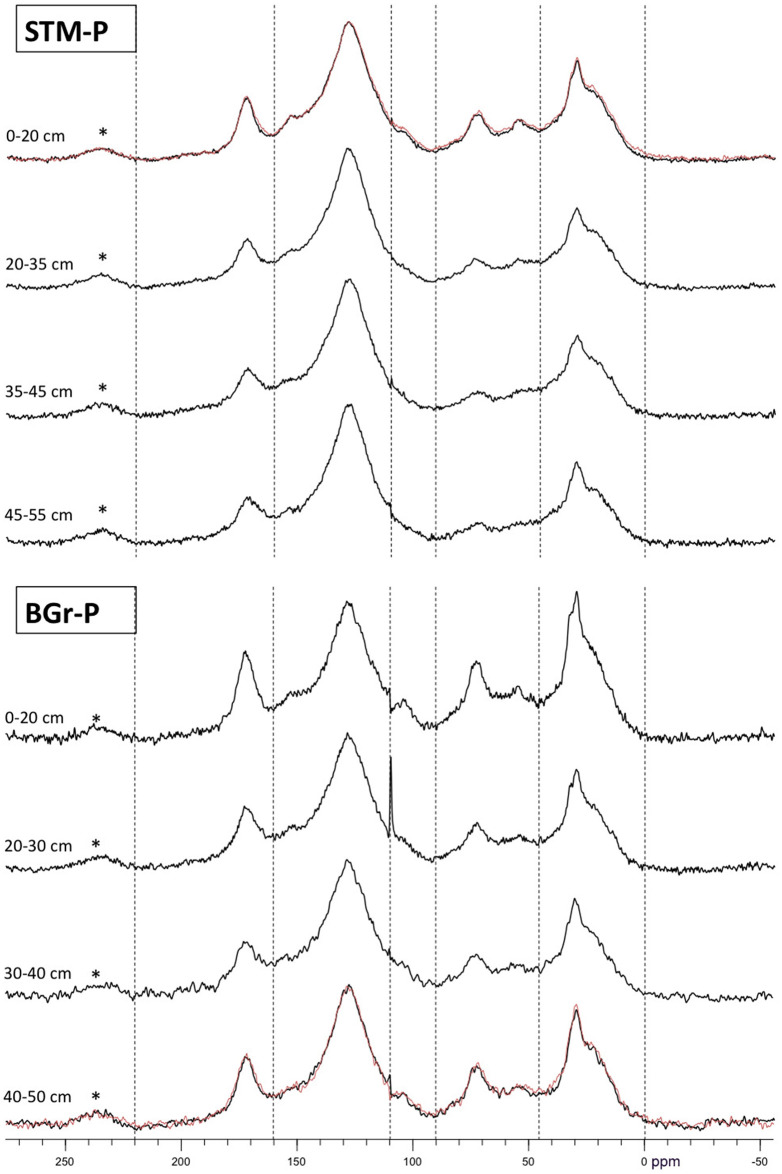
^13^C-CPMAS NMR spectra of HF treated soil samples from four depths at the urban STM-P and extra-urban BGr-P sampling sites. Dashed lines demarcate integration limits as given in Table 1. The overlaid red spectra are repetition measurements to demonstrate reproducibility within each set. Spectra were normalized graphically to the most prominent peak at 129 ppm (* = spinning side bands).

The aromatic C contents of the samples are distributed irregularly within the profiles, but surface soil layers have lower aromatic C content than subsurface layers. Although, the absolute amount of organic matter is lower in the BGr-P site, the relative proportions of carbon species followed the same general sequence on all sites and at all depths: % aromatic C > % alkyl C > % *O/N*-alkyl C ≥ % carboxyl C > % anomeric C (Fig 6). However, the alkyl C/*O/N*-alkyl C ratio increased with depth at the STM-P and SSJG-5 sites. The ratio showed only a slight increase at depth 30–40 cm compared to the top layer at the BGr-P site, while the ratios in the other layers were similar to each other.

## Discussion

### Phenoloxidase activity and soil depth relationships

Differences in surface and subsurface conditions in both terrestrial and aquatic ecosystems—natural or anthropogenic—influence the diversity and variations in their processes and operations. Overall, we observed higher PO enzyme activity in the subsoil relative to the topsoil. Strong relationships between sample depth and various soil-process dependent elements, such as moisture content, organic matter, microbial biomass, and soil respiration were common among all sites (Fig 3). An earlier study by Pind et al. [11] on PO in northern peatlands found that enzymatic activity fell rapidly with depth and was correlated with oxygen availability, which is essential for the catalysis of phenolic compounds (*ortho*-hydroxylation of monophenols to diphenols and oxidation of diphenols to quinones). Low oxygen availability constrains PO-related functionality. The general increase in PO activity detected with depth in our sites, where conditions are drier, i.e., less soil moisture, suggests that oxygen supply was not a limiting factor in this system at the time of enzyme release, and/or there is an abundance of intact enzyme present (Fig 5). These observations suggest that the observed patterns of PO activity were likely in response to factors such as historical substrate availability, input, and accessibility (i.e., organic matter content), rather than oxygen or hydrogen peroxide constraints.

The accumulation of phenolic compounds and subsequent decline in hydrolase activity, both resulting from low PO activity, can impede microbe-driven decomposition in temperate peatlands [5,14]. On our study sites, PO activity increased with depth concomitantly with decreasing organic matter content. ^13^C NMR spectroscopic analysis of C species also revealed that the observed decline in soil organic matter with depth corresponds to decreases in more labile organic carbon fractions as evidenced by changes in the alkyl C/*O/N*-alkyl C ratio of the spectra (Table 1). The observed inverse correlation can be interpreted as a microbial response to an increasingly nutrient poor environment found at lower depths, as it is well established that organic matter content is one of the key controlling variables for enzyme activities [19]. Our analyses demonstrated that organic matter content had the strongest effect on PO activity; a strong negative correlation with organic matter in site SSJG-5 and BGr-P (*R*^*2*^ = 0.91; 0.62; Fig 4). A non-significant correlation for the STM-P site (*R*^*2*^ = 0.37, P > 0.05) was observed. Factors such as lower organic matter content, suboxic or dysoxic conditions, soil mineralogy, and phenolic profiles limit nutrient availability (organic carbon) and accessibility to microorganisms [43–45]. We posit that microorganisms synthesized and released PO enzymes in order to degrade more recalcitrant phenolic compounds that are present in the soil, as evidenced by the dominance of aromatic C structures (Fig 6), as a mechanism to liberate or increase access to labile substrate, and to prevent the inhibition of hydrolytic enzymes involved in microbial metabolism and/or decomposition.

### Relationship between phenoloxidase and microbial activity

The effects of elevated PO activity with depth can also be seen from the pattern of activity of soil microorganisms. Soil microbial biomass and respiration both decreased with depth as expected, however the depth dependent decline in microbial biomass was exponential while soil respiration declined gradually (Fig 3). Whilst adaptation is not entirely dismissed, we propose that it is more plausible that the localised increases in organic carbon availability at the various depths through increased accessibility has led to a rise in the respiration rate of microorganisms; this is proportionally greater at depth relative to surface soil profiles. Nitrogen availability (i.e., low N to C ratio) is known to limit microbial processes, such as, enzyme production, microbial biomass, microbial respiration and ultimately soil organic matter decomposition [46,47]. Although the total nitrogen content decreased with depth across all sample sites, these values were not sub-optimal for microbial growth and associated activities.

The mineralisation of organic matter is generally understood to decline with depth due to reduced energy availability to sustain heterotrophic microbial biomass and their associated activities [43,45]; however, research by Kemmitt et al. [48] on grassland and arable soil showed that the rate of organic matter decomposition was not proportional to the size, activity or composition of the microbial biomass. It was further hypothesised that mineralisation of organic matter is regulated and ‘gated’ by abiotic processes that transform non-bioavailable soil organic matter to bioavailable substrates. For our anthropogenically amended soils, the general decline in microbial biomass and respiration with depth initially suggest a higher decomposition and/or activity rate at the surface than at depth when considered in absolute terms, however when measured in relative term to the biomass size, microorganisms at depth appear to be metabolically more active than their surface counterparts. In addition, the observation of highly active microbial community by mass at depth without an associated increase in community size suggests that the nutrients made available to the system are utilised primarily to maintain functional viability rather than increase biomass and that PO enzyme production is independent of microbial community size. Also of note is the activity of PO in surface and near surface profile samples that receive fresh litter in conditions that are easily available for microorganisms to metabolise. The expression of PO by microbes at these profiles may be seen as mitigation response against the toxicity of phenolic molecules by actively reducing soil phenolic content [6,10].

According to Baldock and Preston [38] and Baldock et al. [39], the ratio of alkyl C to *O/N*-alkyl C can be used as an indicator for assessing the extent of decomposition of organic materials. The ratio of alkyl C to *O/N*-alkyl C increased with depth in sites STM-P and SSJG-5 indicating a higher degree of decomposition at depth than on the surface [40,49]. The ratio remained fairly constant with depth in site BGr-P with only a slightly elevated ratio at 40 cm depth (Table 1). The stagnation in the ratio at BGr-P can be attributed to the higher percentage of clay and silt content (Table 1). Clay minerals are well known to slow decomposition of organic material in soil through the association of SOM with mineral components (adsorption) which in turn reduces substrate availability and/or accessibility to microbes thereby slowing decomposition [43,45,50–52].

### Phenoloxidase enzyme activity in different environments

Phenoloxidase enzymes have been shown in many studies to assume an active role in a range of biogeochemical processes in different ecosystems [5,11,14,53–55]. Phenoloxidase activity is not determined by a single factor but by multiple variables (moisture, aeration, organic matter, pH, Fe^2+^, phenolic content) depending on the physicochemical profile of each system. Kang et al. [19] examined the rate of microbial enzyme activity including PO in tropical marsh soils with different water regimes to determine mechanisms associated with decomposition in such ecosystems. They found that enzyme activities were higher in waterlogged soils than in dry soils, however, this effect was attributed to higher nutrient and organic matter availability in the wet soils than in drier soils. They also demonstrated that depth had no discernible effect on enzyme activity and that water supply was a limiting factor in the system although only two soil depths (0–5 cm and 5–10 cm) were studied. Similarly, Liu et al. [56] examined the effects of flooding on soil phenoloxidase activity during rice straw decomposition and concluded that enzymatic activity was higher in flooded soils than non-flooded soils.

These results are contradictory to previous reports on enzyme activity in temperate marsh and peatlands where water-table drawdown stimulates enzyme activity [54,57]; however, the studies by Kang et al. [19] and Liu et al. [56] demonstrate that the oxygen content of the systems was not sub-optimal for enzyme activity and therefore not a limiting factor in the wet system, and that nutrient and organic matter content constrained enzyme activity in the dry system. Furthermore, Williams et al. [55] investigated the phenoloxidase activity in *Sphagnum* and *Carex* wetland in New York. They reported that pH and phenolic contents had more effect on phenoloxidase activity than aeration. In our study sites, the soil pH ranges are not sub-optimal for phenol oxidase enzyme activity [11,41]. However, the patterns of phenoloxidase enzyme activity observed show a closer relationship with soil organic carbon content and speciation, than other factors investigated (soil depth, aeration, pH, and microbial abundance).

## Conclusion

Lower organic matter content and a corresponding decrease in the soil labile (*O/N-alkyl C*) fraction was a good predictor of PO activity in the historic Anthrosols. We also demonstrate for the first time that an enhanced presence of PO may play a major role in the nutrient dynamics in such modified soils. Historical practices saw high inputs of diverse organic-rich materials to soils. The mixing in of these materials of diverse origin would have elevated the concentrations of phenolic compounds in such anthrosols above background levels. Phenoloxidase enzymes are then synthesised and deployed by soil microorganisms to counter the deleterious effects of phenolic compounds on general enzyme and microbial activity within the soil. Further studies are needed to determine the abundance and complexity of phenolic compounds *in situ*, as well as microbial community structure, to better understand the effects of substrate concentration and the associated microbial response.

## Supporting information

S1 Table Relative contributions of carbon species to the total signal intensity in ^13^C NMR spectroscopy of soil samples in study sites. (DOCX)

S2 Table Soil chemical properties. (DOCX)

S1 Data Data (underpinning the study). (XLSX)
