# Supplementary material for: Phenoloxidase activity and organic carbon dynamics in historic Anthrosols in Scotland, UK
Source: PLoS One. 2021 Oct 27;16(10):e0259205. doi: 10.1371/journal.pone.0259205 (PMC8550383; doi:10.1371/journal.pone.0259205)
Supplement: S1 Table — (DOCX) [file pone.0259205.s001.docx]

**S1 Table. Relative contributions of carbon species to the total signal intensity in ^13^C NMR spectroscopy of soil samples in study sites.**

| Sample Location & Sample Depth (cm) | % Alkyl C (0-45 ppm) | % O/N-Alkyl C (45-90 ppm) | % Anomeric C (90-110 ppm) | % Aromatic C (110-160 ppm) | % Carboxyl C (160-220 ppm) | Alkyl C  O/N-Alkyl C | % Clay (%) | Silt (%) |
| --- | --- | --- | --- | --- | --- | --- | --- | --- |
| **Medieval Urban Centre** |  |  |  |  |  |  |  |  |
| **South Street (SST-1)**  0-20 | 22 | 17 | 4 | 45 | 12 | 1.29 | 4.1 | 64.0 |
| 20-30 | 20 | 11 | 3 | 54 | 11 | 1.82 | 6.0 | 71.0 |
| 30-40 | 21 | 12 | 4 | 53 | 10 | 1.75 | 11.0 | 89.0 |
| 40-50 | 22 | 15 | 4 | 48 | 11 | 1.47 | 10.1 | 89.9 |
| Extra Urban Site |  |  |  |  |  |  |  |  |
