# Supplementary material for: Phenoloxidase activity and organic carbon dynamics in historic Anthrosols in Scotland, UK
Source: PLoS One. 2021 Oct 27;16(10):e0259205. doi: 10.1371/journal.pone.0259205 (PMC8550383; doi:10.1371/journal.pone.0259205)
Supplement: S2 Table — (DOCX) [file pone.0259205.s002.docx]

**S2 Table. Soil chemical properties**

| Sample | Depth (cm) | Total N (%) | C/N ratio | pH (CaCl_2_) |
| --- | --- | --- | --- | --- |
| STM-P | 12 | 0.28 | 18.54 | 5.58 |
|  | 30 | 0.32 | 13.56 | 5.92 |
|  | 48 | 0.23 | 12.30 | 6.06 |
|  | 71 | 0.12 | 21.75 | 5.70 |
|  | 87 | 0.17 | 23.65 | 5.89 |
| SSJG-5 | 0-20 | 0.85 | 9.67 | 5.48 |
|  | 20-30 | 0.62 | 12.66 | 5.80 |
|  | 30-40 | 0.39 | 17.26 | 5.84 |
|  | 40-50 | 0.50 | 14.50 | 5.91 |
|  | 50-65 | 0.39 | 17.31 | 5.77 |
|  | 65-75 | 0.39 | 17.31 | 5.84 |
|  | 75-85 | 0.36 | 12.42 | 5.71 |
|  | 85-100 | 0.23 | 13.00 | 5.66 |
| BGr-P | 10 | 0.51 | 4.55 | 5.58 |
|  | 23 | 0.36 | 9.19 | 5.95 |
|  | 46 | 0.26 | 9.35 | 5.61 |
|  | 60 | 0.11 | 4.09 | 5.84 |
